# Laparoscopy in Low-Income Countries: 10-Year Experience and Systematic Literature Review

**DOI:** 10.3390/ijerph18115796

**Published:** 2021-05-28

**Authors:** Damiano Pizzol, Mike Trott, Igor Grabovac, Mario Antunes, Anna Claudia Colangelo, Simona Ippoliti, Cristian Petre Ilie, Anne Carrie, Nicola Veronese, Lee Smith

**Affiliations:** 1 Italian Agency for Development Cooperation, Khartoum 11111, Sudan; 2 The Cambridge Centre for Sport & Exercise Sciences, Anglia Ruskin University, Cambridge CB1 1PT, UK; mike.trott@pgr.anglia.ac.uk (M.T.); lee.smith@aru.ac.uk (L.S.); 3 Vision and Eye Research Institute, Anglia Ruskin University, Cambridge CB1 1PT, UK; 4 Department of Social and Preventive Medicine, Centre for Public Health, Medical University of Vienna, Vienna 1010, Austria; igor.grabovac@meduniwien.ac.at; 5 Department of Surgery, Central Hospital of Beira, Beira 13016, Mozambique; majomantu@gmail.com; 6 Department of Surgery, Catholic University of Mozambique, Beira 13016, Mozambique; 7 Department of Surgery and Organ Transplantation, University of Padua, 35125 Padua, Italy; colangeloannaclaudia@gmail.com; 8 Department of Urology, The Queen Elizabeth Hospital, King’s Lynn PE30 4ET, UK; ippoliti.simona@gmail.com (S.I.); Petre-Cristian.Ilie@qehkl.nhs.uk (C.P.I.); AnneMarie.Carrie@qehkl.nhs.uk (A.C.); 9 Geriatric Unit, Department of Internal Medicine and Geriatrics, University of Palermo, 90121 Palermo, Italy; ilmannato@gmail.com

**Keywords:** laparoscopy, low-income countries, minimal invasive surgery

## Abstract

Laparoscopy is a procedure that ultimately reduces hospital stay time and speeds up post-operative recovery. It is mainly performed in high-income countries but its implementation in many low- and middle-income countries (LMICs) is increasing. However, no aggregate data exist regarding the outcomes of this procedure in resource-limited settings. We retrospectively reviewed all cases of laparoscopy recorded from January 2007 to March 2017 at the Department of Surgery of Beira to assess the related outcomes. Moreover, we performed a systematic review of the laparoscopic practices and outcomes in low-income countries. Data from the Department of Surgery of Beira identified 363 laparoscopic procedures, mainly relating to gynecological diseases, cholelithiasis, and appendicectomy with only a 1.6% complication rate (6 cases) and a 1.9% conversion rate (7 cases) to open surgery. The systematic review showed a pooled risk of overall complications significantly lower in laparoscopic vs. open appendicectomy (OR = 0.43; 95% CI 0.19–0.97; I^2^ = 85.7%) and a significantly lower risk of infection (OR = 0.53; 95% CI 0.43–0.65; I^2^ = 0.00%). The pooled SMD in operation duration in laparoscopic vs. open appendectomy was 0.58 (95% CI −0.00; 1.15; I^2^ = 96.52), while the pooled SMD in hospitalization days was −1.35 (95% CI −1.87; −0.82; I^2^ = 96.41). Laparoscopy is an expensive procedure to adopt as it requires new equipment and specialized trained health workers. However, it could reduce post-operative costs and complications, especially in terms of infections. It is crucial to increase its accessibility, acceptability, and quality particularly in LMICs, especially during this COVID-19 era when the reduction of patient hospitalization is essential.

## 1. Introduction

Laparoscopy is defined as a type of surgical procedure that allows medical doctors to access the abdomen, pelvis, or thorax through small incisions on the skin [1]. The main advantages are: (I) to shorten the hospital stay and the recovery time, (II) to reduce pain and post-surgery bleeding, and (III) to minimize scarring [1]. Laparoscopy can be used to perform both diagnostic and treatment procedures, and it is mainly used in gynecology, gastrointestinal surgery, and urology [1]. Some of the complications regarding this procedure are related to cavity access and the possibility of developing physiologic pneumoperitoneum. Moreover, in complicated cases, it may require a conversion to an open procedure [2]. Minor complications, including minor bleeding and bruising around the incision, infections, nausea, and vomiting are estimated to occur in about 2% of the cases [1]. Major complications, occurring in 1 out 1000 cases, may result from damage to an organ or a major artery, or complications such as gas embolism may arise from the use of carbon dioxide during the procedure [1]. The laparoscopic approach is preferred for a number of surgical procedures in high-income countries (HICs), while it is still not available in many low- and middle-income countries (LMICs) due to the high cost of purchasing and maintaining the equipment, and the lack of trained surgeons [3]. The equipment costs are not the only limit for implementing laparoscopy in LMICs. Appropriate training is also difficult due to the lack of dry and wet lab facilities and unaffordable trained specialists [4]. Moreover, in many LMICs it is difficult to promote new ideas in surgery, not only among patients but also among local surgeons due to cultural and social barriers [5]. However, initiatives are being implemented in LMICs in order to train dedicated health workers and to promote laparoscopy, especially in order to minimize post-surgical infection and to reduce recovery time [6,7]. More importantly, the advantages of laparoscopy compared to open surgery could be even more evident in settings with limited access to blood transfusion, clean water, and poor healthy living conditions [8]. Moreover, diagnostic laparoscopy may also be more economical and clinically effective in LMICs considering the lack of modern diagnostic imaging [9]. Despite the growing body of literature on laparoscopy in LMICs, no aggregate data exist regarding the outcomes of this procedure in resource-limited settings.

The aim of the present study was to assess the outcomes of laparoscopy in Beira, Mozambique over a period of 10 years and compare them to global outcomes in order to consider if laparoscopy could be introduced in the local setting without increasing complication rates, operation time, and hospital stay. This paper presents a case series of laparoscopic procedures performed from 2007 to 2017 at the Central Hospital of Beira (CHB) and carries out a systematic review of laparoscopy outcomes in LMICs.

## 2. Materials and Methods

### 2.1. Case Series

#### 2.1.1. Setting

The city of Beira has approximately 500,000 inhabitants, 17% of which are less than 5 years old. The CHB is a 1020-bed government tertiary referring and teaching hospital for the central region (population approximately 7 million) of Mozambique, and the second-largest hospital in the country. The CHB Department of Surgery consists of six specialists and is a landmark for the whole city of Beira and the province of Sofala.

#### 2.1.2. Data Collection and Analysis

Data registers of CHB’s Department of Surgery were retrospectively reviewed to identify all cases of laparoscopic surgery from January 2007 to December 2017. The “Laparoscopy register” is a dedicated book filled in by the surgeon after each intervention. The lack of a standard and predefined page structure led to some missing data, but all present data are fully reliable. The extracted data provided a database with general information, organized in the following variables: gender, age, HIV status diagnosis, American Society of Anesthesiologists (ASA) physical status classification, operative duration, complications, and conversion to open surgery. We conducted a descriptive analysis of all collected data.

### 2.2. Systematic Review

This systematic review adhered to the PRISMA [10] and MOOSE [11] statements and followed a structured protocol available under reasonable request from the corresponding author.

#### 2.2.1. Search Strategy

Two investigators (MT and DP) independently conducted a literature search using the MEDLINE/PubMed, Scopus, CINAHL, Embase PsycINFO, and Cochrane Library databases, from the date of inception to 9 November 2020. The following search strategy was used: (Laparoscopy OR Laparoscopic OR Laparoscopic surgery OR minimally invasive surgery), and (LMIC code OR low and middle-income count* OR low-resource settings OR developing countries), and (Safety OR Costs and cost analysis OR Outcome OR Mortality OR Morbidity OR Length of stay OR Complications). The references of retrieved articles together with the proceedings of relevant conferences were hand-searched in order to identify other potentially eligible studies for inclusion in the analysis missed by the initial search, or to find any unpublished data.

The literature search, assessment of inclusion and exclusion criteria, quality of studies, and extraction of data were independently undertaken and verified by two investigators (MA, DP). The results were then compared, and in the case of discrepancies, a consensus was reached with the involvement of a third senior investigator (LS). There was no language restriction.

#### 2.2.2. Type of Studies, Inclusion and Exclusion Criteria

Following the PICOS (participants, intervention, controls, outcomes, study design) criteria, we included studies assessing:

P: Patients who underwent laparoscopic procedures;

I: Laparoscopy;

C: Patients who underwent open procedures;

O: Procedure duration, complications, morbidity, length of stay;

S: Observational (case-control, cross-sectional).

All retrospective or prospective studies reporting laparoscopic procedures in low-income countries were included. Studies were excluded if they had no data on outcomes of laparoscopic procedures. No language restriction was placed.

#### 2.2.3. Data Extraction and Statistical Analyses

For each eligible study, two independent investigators (NV, DP) extracted: name of the first author and year of publication, setting, sample size, mean age of the population, % of females, operative duration, blood loss, ASA classification, days of hospitalization, number of laparoscopies converted to open surgery, and complications (infection, duodenal injury, hernia, nausea and vomiting, fever and pain).

#### 2.2.4. Outcomes

The main outcome was the comparison between laparoscopy and open surgery in terms of complications (especially infection), operative time, and hospitalization time.

#### 2.2.5. Assessment of Quality of Studies

Two independent authors (DP, SI) assessed the quality of studies using the Newcastle–Ottawa Scale (NOS) [12]. The NOS assigns a maximum of 9 points, based on three quality parameters: selection, comparability, and exposure and outcome for case-control and cross-sectional studies, respectively. According to the NOS grading in past reviews, we graded studies as having a high (<5 stars), moderate (5–7 stars), or low risk of bias (≥8 stars) [13].

#### 2.2.6. Data Synthesis and Statistical Analysis

Due to heterogeneity, a random-effects model was conducted using the method proposed by Der Simonian and Laird [14], weighting cases using the inverse of the variance, calculating either the prevalence rates with 95% confidence intervals (CIs), or the odds ratios (ORs) using the Comprehensive Meta-Analysis Version 3 [15], with the aim of calculating: (I) the risk of adverse events for laparoscopic vs. open surgery, (II) the prevalence of adverse events following laparoscopic surgery, and (III) the differences in hospitalization time and operation duration for laparoscopic vs. open surgery.

The meta-analysis was conducted in the following steps: 1. ORs were calculated with 95% CIs using sample sizes and the number of adverse events in laparoscopic vs. open surgery, or prevalence rates of adverse events were calculated with 95% CIs using total ns and event ns. 2. Heterogeneity was assessed with the I^2^ statistic for all analyses, with 0–50% being classified as low, >50–75% moderate, and >75% high heterogeneity [16]. 3. Meta-regression analyses were performed on potential moderators of adverse events, including mean age and sex (where data were available). 4. As recommended by Sterne and colleagues [17], if the meta-analysis exceeded 10 studies, publication bias was assessed with the Begg–Mazumdar Kendall’s tau [18] and the Egger bias test [19]). If publication bias was found to adjust for potential publication bias, the trim-and-fill adjusted analysis was used to remove the most extreme small studies from the positive side of the funnel plot, and effect sizes were re-calculated until the funnel plot was symmetrical with the new effect size [20].

### 2.3. Ethical Statement

The Clinical Board of Beira Central Hospital approved the study and granted the use of anonymized data for scientific purposes. The Clinical Board waived the need for written informed consent given the retrospective nature of the study and the use of anonymized data from hospital records.

## 3. Results

### 3.1. Case Series

A total of 363 laparoscopies were performed at the Department of Surgery following international standard guidelines at the CHB between January 2007 and December 2017, Table 1.

All procedures were performed by two surgeons, the only surgeons at the CHB who had received international training and were able to carry out this procedure. Among the patients, 307 (84.6%) were female, mean age 37.7 (range 16–79), and 56 (15.4%) were male, mean age 48.6 (range 14–72). The HIV status was available only in 25 cases, with a prevalence of 72%. The main reason for laparoscopy (169 cases) was gynecological procedures. Among these, 149 were diagnostic, performed to investigate infertility, looking for adherences, endometriosis lesions, uterine malformations, cystic lesions, salpingitis, and tubal patency. The remaining were due to clinical suspicion of endometriosis (13 cases), ovarian cancer (4), and ectopic pregnancy (3). The other reasons for laparoscopy were cholelithiasis (43.2%), appendicectomy (5%), genitourinary, laryngeal, and liver diseases (1.6, 1.4, and 1.4%, respectively).

In Table 2 we reported data regarding operative procedures.

The majority of patients were classified as ASA II (56.6%), followed by ASA I (39.3%). A total of 76.9% of interventions were performed in less than 120 min and only 5.5% required more than 180 min. Only six cases of cholecystectomy (1.6%) had complications and it was due to the Mirizzi Syndrome and an excess of stones (two each), gallbladder and cystic artery clip. Only seven (1.9%) were converted to open surgery.

### 3.2. Systematic Review

#### 3.2.1. Literature Search

As shown in Supplementary Figure S1, we initially found 654 possible eligible articles. After removing 570 papers through the title/abstract screening, 84 were retrieved as full text. Out of the 84 full-text articles retrieved, 55 satisfied the inclusion/exclusion criteria and were included in the systematic review and meta-analysis.

#### Excluded Studies

Among the relevant studies, 29 failed to meet the inclusion criteria and were excluded from this review, mainly due to the lack of data on laparoscopy, the description of procedures, mini-laparotomy, reference to complications, or laparoscopy used as support to open surgery.

#### 3.2.2. Risk of Adverse Events for Laparoscopic vs. Open Surgery

**Figure 1:**
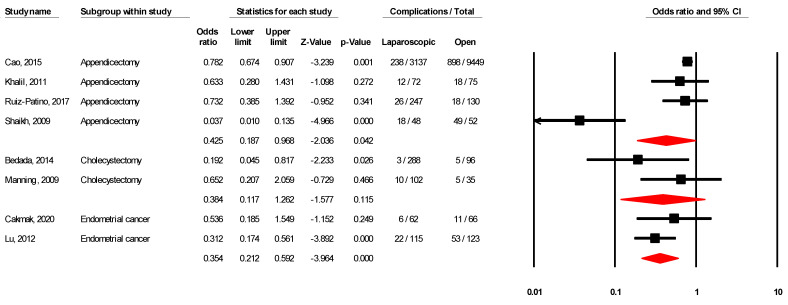
Forest plot for overall complications in laparoscopic vs. open surgery.

As shown in Table 3 and Figure 1, the pooled risk of overall complications was significant in laparoscopic vs. open appendicectomy (OR = 0.43; 95% CI 0.19–0.97; I^2^ = 85.7%), significant in endometrial cancer (OR = 0.35; 95% CI 0.21–0.59; I^2^ = 0.00%), and non-significant in both cholecystectomy (OR = 0.38; 95% CI 0.12–1.26; I^2^ = 40.56%).

**Figure 2:**
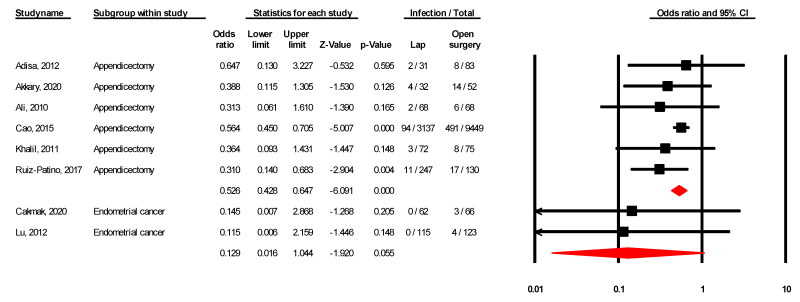
Forest plot for risk of infection in laparoscopic vs. open surgery.

The pooled risk of infection was significant in laparoscopic vs. open appendicectomy (OR = 0.53; 95% CI 0.43–0.65; I^2^ = 0.00%), and non-significant in endometrial cancer (OR = 0.13; 95% CI 0.02–1.04; I^2^ = 0.00%), see Figure 2.

#### 3.2.3. Prevalence of Adverse Outcomes in Laparoscopic Surgery

**Figure 3:**
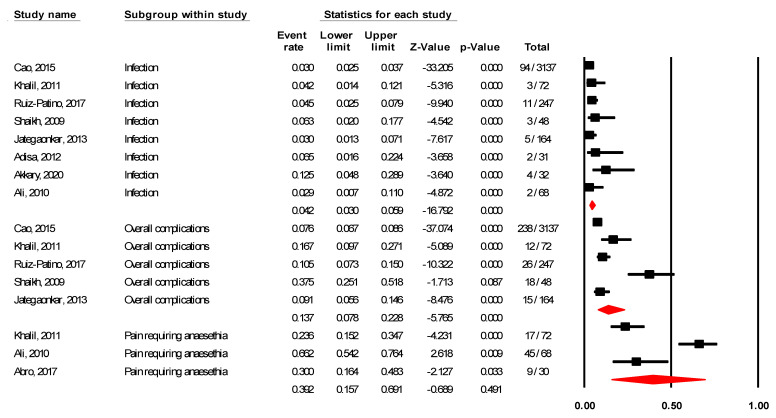
Prevalence of adverse events in laparoscopic appendectomy.

As reported in Table 4, regarding laparoscopic appendectomy, the prevalence of overall complications was 13.7% (95% CI 7.8–22.8%; I^2^ = 91.93%), prevalence of infection was 4.2% (95% CI 3.0–5.9%; I^2^ = 38.95%), and prevalence of pain requiring analgesia was 39.2% (95% CI 15.7–69.1%; I^2^ = 92.36%), see Figure 3.

**Figure 4:**
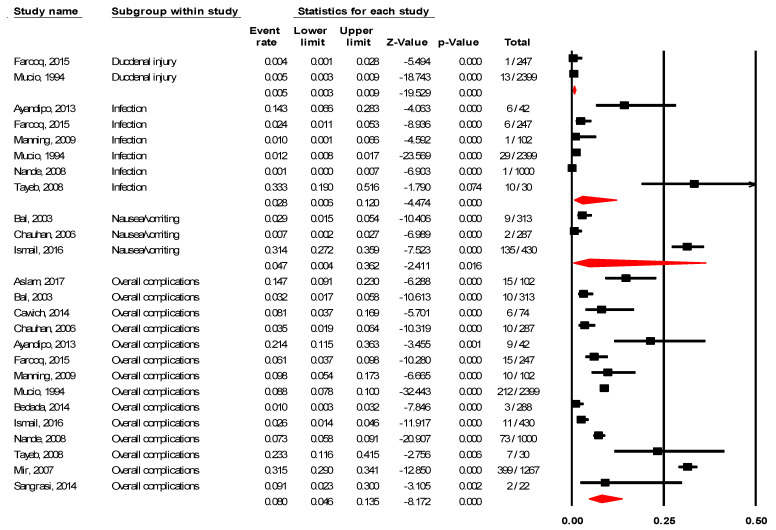
Prevalence of adverse events in laparoscopic cholecystectomy.

In laparoscopic cholecystectomy, the prevalence of overall complications was 8.0% (95% CI 4.6–13.5%; I^2^ = 97.40%), prevalence of infection was 2.8% (95% CI 0.6–12.0%; I^2^ = 95.18%), prevalence of duodenal injury was 0.5% (95% CI 0.3–0.9%; I^2^ = 0.00%), and prevalence of nausea/vomiting was 4.7% (95% CI 0.4–36.2%; I^2^ = 97.78%), see Figure 4.

#### 3.2.4. Hospitalization Days and Operation Duration for Laparoscopic vs. Open Appendectomy

**Figure 5:**
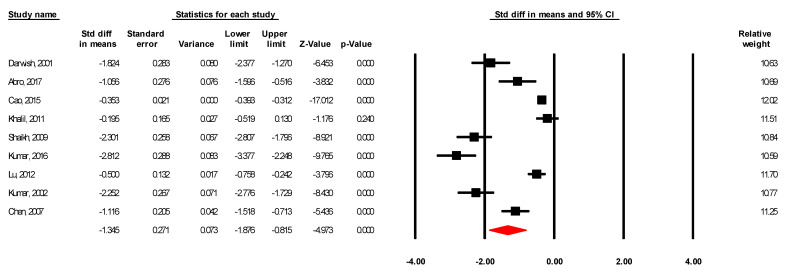
Standard mean differences in hospitalization days for laparoscopic vs. open appendectomy.

**Figure 6:**
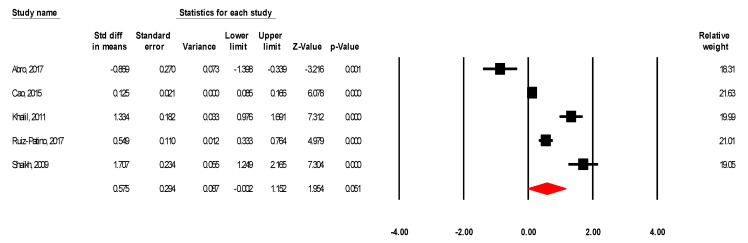
Standard mean differences in operation duration for laparoscopic vs. open appendectomy.

Only the results for appendectomy yielded enough data to be meta-analyzed. The pooled SMD in hospitalization days for laparoscopic vs. open appendectomy (see Figure 5) was −1.35 (95% CI −1.87; −0.82; I^2^ = 96.41), and the pooled SMD in operation duration for laparoscopic vs. open appendectomy (see Figure 6) was 0.58 (95% CI −0.00; 1.15; I^2^ = 96.52).

#### 3.2.5. Publication Bias

No included outcome suffered publication bias.

#### 3.2.6. Quality of Studies

The overall mean score of the studies was 5.2 (range: 4–7), indicating an overall satisfactory quality, according to the NOS (Supplementary Table S1).

## 4. Discussion

The laparoscopic approach is an increasingly common procedure in LMICs, owing to more training activities and support programs that secure the required equipment for the operation [21]. In the studied hospitals, more than 350 laparoscopic surgeries both for diagnostic and therapeutic purposes were performed in a span of ten years. This number would have likely been higher if the COVID-19 pandemic had not reduced the number of surgeries, allowing only urgent open procedures; Cyclone Idai also destroyed sections of the registers, thus resulting in missing data. Our cases mainly included gynecological diseases, cholelithiasis, and appendectomy that are also the main reasons for laparoscopy in other LMIC studies. HIV status was reported in 25 cases and more than 70% of these cases were positive. Although these data do not reflect the HIV prevalence in the general population, Mozambique has one of the highest incidence of HIV worldwide, with an estimated prevalence in adults between 15 and 49 years of 12.5%; HIV is one of the main causes of morbidity and mortality, especially in children [21]. This aspect, which is not covered by any of the 55 studies included in the systematic review, is one of the major arguments for supporting the application of laparoscopy in HIV-endemic areas; the reduced intra- and post-operative bleeding, and its potentially lower risk for health workers makes it the ideal medical procedure. Moreover, considering the COVID-19 pandemic, the laparoscopy approach represents a safer procedure for surgeons as it is performed in a closed cavity, enabling the containment of aerosols [22].

The American Society of Anesthesiologists Classification includes the following six classes: (I) normal, healthy patients, (II) patients with mild systemic diseases, (III) patients with severe systemic diseases, incapacitating but not life threatening, (IV) patients with severe systemic diseases that are a constant threat to life, (V) moribund patients who are not expected to survive without an operation, and (VI) declared brain-dead patients [23]. Interestingly, the laparoscopic approach allowed surgery in patients belonging to ASA classes III and IV—those who likely could not undergo open surgery. In the majority of the patients (76.9%), the procedure was performed in less than 2 h, while only 5 cases took more than 3 h. The time of surgery is influenced by the fact that the majority of the cases were diagnostic procedures. Data from the systematic review suggest that, in general, the amount of time required for laparoscopy is greater than that required for open surgery, but the hospitalization period is significantly lower for laparoscopy, highlighting the benefit of the minimal invasive approach. This is especially significant in a limited-resource setting where the hygienic conditions are generally poor. In our series, the intra-operative complication rate and the necessity to convert the procedure from laparoscopy to open surgery were very low, with six (1.6%) and seven (1.9%) cases, respectively. We believe that the relatively low conversion rate is influenced by the case mix, with the majority being diagnostic and selected cases. We would initially expect this to increase if more highly complex cases were managed, which would lead to a subsequent reduction of conversion rates after the learning curve for that particular procedure is passed. Our data are similar to those reported in literature, and the meta-analysis data showed significantly higher complication rates in open surgery than in laparoscopy. Interestingly, this was found in all groups of patients, and it was particularly evident for infections. The hospitalization period is particularly crucial in LMICs, considering the overall household conditions. It makes the laparoscopy procedure the ideal choice wherever possible. Moreover, it is also suitable in terms of fighting antibiotic resistance, reducing prescriptions in settings with general low adherence to therapy and frequent misuse [24]. We acknowledge a potential selection bias, as it might have been a tendency to select fitter patients for the laparoscopic approach, with more complicated cases still being treated with open surgery. This trend will potentially be reversed with time and experience.

The combination of a ten-year novel case series and meta-analyses is a clear strength of this study. However, findings from this study should be interpreted in light of its limitations. First, our study was retrospective and it was not possible to compare the results of laparoscopy with similar open procedures using a matched cohort of patients. Second, it was not possible to extend the follow-up beyond the primary hospital stay in order to assess long-term complications. Third, there was only partial data of the case series, mainly due to Cyclone Idai. Finally, there was a lack of clinical data both in this study and in the literature review, thus preventing more conclusive results; the quality of included studies likewise showed just a satisfactory quality, with a mean 5.2 NOS score.

## 5. Conclusions

In conclusion, laparoscopy could be introduced in low-resource settings without increasing complication rates, operation time, and hospital stay. In fact, although laparoscopy is an expensive procedure requiring adequate equipment and specialized well-trained health workers, it could reduce the post-operative costs and complications. In particular, in limited-resource settings with generally poor hygienic conditions, it could be effective in reducing infections and thus, in fighting antibiotic resistance. Although more effective efforts should be put in place in order to increase its accessibility, acceptability, and quality, laparoscopic surgery should be considered safe, effective, and feasible also in LMICs, especially in this COVID-19 era, during which it is essential to reduce the hospitalization of patients.

## Figures and Tables

**Table 1:** General information and diagnosis.

Variables	*N* (%)
Number of cases	363
Gender	
Female	307 (84.6%)
Male	56 (15.4%)
Age	
Mean age	39.4
Range age	14–79
Diagnosis	
Gynecological diseases	169 (46.6%)
Cholelithiasis	157 (43.2%)
Appendicectomy	18 (5%)
Genito-urinary diseases	6 (1.6%)
Laryngeal diseases	5 (1.4%)
Liver diseases	4 (1.1%)
Missing data	4 (1.1%)

**Table 2:** Surgery information.

Variables	N (%)	Missing Data *N* (%)
ASA classification		73 (20.1)
I	114 (39.3%)	
II	164 (56.6%)	
III	7 (2.4%)	
IV	5 (1.7%)	
Time of surgery		272 (74.9)
<2 h	70 (76.9%)	
>2 and <3 h	16 (17.6)	
>3 h	5 (5.5%)	
Intraoperative complications		0 (0)
Yes	6 (1.6%)	
No	357 (98.4%)	
Conversion to open surgery		0 (0)
Yes	7 (1.9%)	
No	356 (98.1%)	

ASA = American Society of Anesthesiologists, physical status classification.

**Table 3:** Risk of adverse events for laparoscopic vs. open surgery.

Outcome	Procedure Type		Laparoscopy		Open Surgery		Meta-Analysis			Heterogeneity
		Number of Studies	Total *n*	Total Events	Total *n*	Total Events	Odds Ratio (95% CI)	*p*-Value	Difference between Groups	I^2^
Overall complications	Appendectomy	4	3504	294	9706	983	0.425 (0.187–0.968)	0.042	*p =* 0.934	85.728
	Cholecystectomy	2	390	13	131	10	0.384 (0.117–1.262)	0.115		40.557
	Endometrial cancer	2	177	28	189	64	0.354 (0.212–0.592)	<0.001		0.000
Infection	Appendectomy	6	3587	116	9857	544	0.526 (0.428–0.647)	<0.001	*p =* 0.190	0.000
	Endometrial cancer	2	177	0	189	7	0.129 (0.016–1.044)	0.055		0.000

**Table 4:** Prevalence of adverse outcomes in laparoscopic appendectomy and cholecystectomy.

Procedure Type	Outcome				Meta-Analysis		Heterogeneity
		Number of Studies	Total *n*	Total Events	Prevalence	95% CI	I^2^
Appendectomy	Overall complications	5	3668	309	13.7%	7.8–22.8%	91.93
	Infection	8	3799	116	4.2%	3.0–5.9%	38.95
	Pain requiring analgesia	3	140	71	39.2%	15.7–69.1%	92.36%
Cholecystectomy	Overall complications	14	6603	767	8.0%	4.6–13.5%	97.40%
	Infection	6	3820	553	2.8%	0.6–12.0%	95.18%
	Duodenal injury	2	2646	14	0.5%	0.3–0.9%	0.00%
	Nausea/vomiting	3	1030	146	4.7%	0.4–36.2%	97.78%

## Data Availability

All data are included in the manuscript.

## Supplementary Materials

The following are available online at https://www.mdpi.com/article/10.3390/ijerph18115796/s1, Figure S1: PRISMA flow-chart, Table S1: The quality of the studies according to the Newcastle - Ottawa assessment Scale.
