# Supplementary material for: Laparoscopy in Low-Income Countries: 10-Year Experience and Systematic Literature Review
Source: Int J Environ Res Public Health. 2021 May 28;18(11):5796. doi: 10.3390/ijerph18115796 (PMC8198316; doi:10.3390/ijerph18115796)
Supplement: Supplementary file 1 [file ijerph-18-05796-s001.zip › ijerph-1191329-supplementary.pdf]

Supplementary Materials

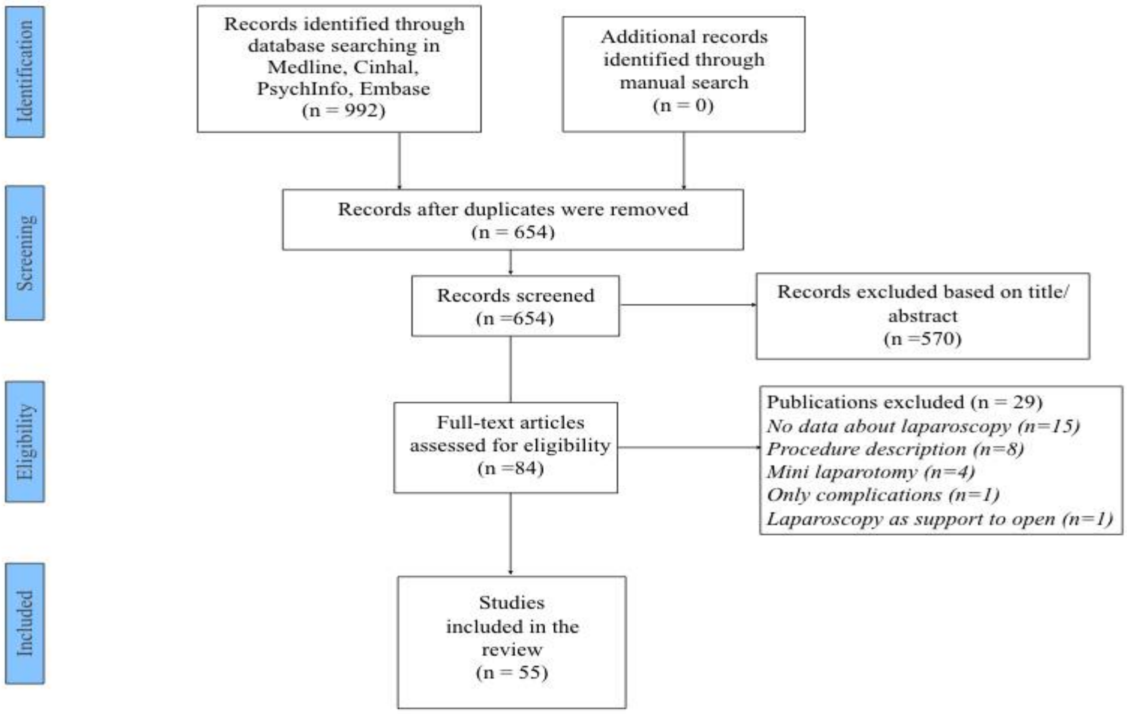

Figure S1. PRISMA flow-chart.

Table S1. The quality of the studies according to the Newcastle - Ottawa assessment Scale.

**Case- control**	**Selection**				**Comparability**	**Exposure**			**Tot**
Abro, 2017 [1]	1	0	0	1	1	1	1	0	5
Adisa, 2012 [2]	1	1	0	1	1	1	1	0	6
Akkary, 2020 [3]	1	0	0	1	1	1	1	0	5
Ali, 2010 [4]	1	1	0	1	1	1	1	0	6
Ali, 2018 [5]	1	1	0	1	1	1	1	0	6
Bedada, 2014 [6]	1	1	0	1	2	1	1	0	7
Cakmak, 2020 [7]	1	1	0	1	1	1	1	0	6
Cao, 2015 [8]	1	1	0	1	2	1	1	0	7
Chen, 2007 [9]	1	0	0	1	1	1	1	0	5
Darwish, 2001 [10]	1	1	0	0	1	1	1	0	5
Hamamci, 2002 [11]	1	0	0	0	1	1	1	0	4
Khalil, 2011 [12]	1	1	0	1	1	1	1	0	6

Kumar, 2002 [13]	1	0	0	0	1	1	1	0	4
Kumar, 2016 [14]	1	1	0	1	1	1	1	0	6
Lu, 2012 [15]	1	1	0	1	1	1	1	0	6
Mahadevappa, 2016 [16]	1	1	0	0	1	1	1	0	5
Mahmoud, 2017 [17]	1	1	0	0	1	1	1	0	5
Manning, 2009 [18]	1	1	0	1	1	1	1	0	6
Ruiz-Patino, 2017 [19]	1	1	0	1	1	1	1	0	6
Shaikh, 2009 [20]	1	0	0	1	1	1	1	0	5
Tunruttanakul, 2020 [21]	1	1	0	1	1	1	1	0	6
**Cross-sectional**	**Selection**				**Comparability**	**Outcome**			**Tot**
Amin, 2015 [22]	1	0	0	1	1	1	1		5
Aslam, 2017 [23]	1	0	0	1	1	1	1		5
Ayandipo, 2013 [24]	1	0	0	1	0	1	1		4
Bal, 2003 [25]	1	1	0	1	1	1	1		6
Balogun, 2020 [26]	1	0	0	1	1	1	1		5
Benkabbou, 2015 [27]	1	0	0	1	0	1	1		4
Cawich, 2014 [28]	1	1	0	1	1	1	1		6
Chauhan, 2006 [29]	1	0	0	1	1	1	1		5
Dalvi, 2005 [30]	1	0	0	1	0	1	1		4
Dan, 2010 [31]	1	0	0	1	1	1	1		5
Farooq, 2015 [32]	1	0	0	1	1	1	1		5
Gupta, 2016 [33]	1	0	0	1	0	1	1		4
Hannan, 2012 [34]	1	0	0	1	0	1	1		4
Ismail, 2016 [35]	1	1	0	1	2	1	1		7
Jategaonkar, 2013 [36]	1	0	0	1	1	1	1		5
Karim, 2017 [37]	1	1	0	1	1	1	1		6
Kumar, 2004 [38]	1	0	0	1	1	1	1		5
Kumar, 2009 [39]	1	0	0	1	1	1	1		5
Mahdi, 2015 [40]	1	0	0	1	1	1	1		5
Mboudou, 2014 [41]	1	1	0	1	1	1	1		6
Mir, 2007 [42]	1	1	0	1	1	1	1		6
Mir, 2009 [43]	1	0	0	1	1	1	1		5
Misauno, 2012 [44]	1	0	0	1	0	1	1		4
Mishra, 2020 [45]	1	0	0	1	1	1	1		5
Mucio, 1994 [46]	1	0	0	1	1	1	1		5
Nande, 2002 [47]	1	0	0	1	1	1	1		5

O'Connor, 2017 [48]	1	0	0	1	1	1	1	5
Pareja, 2012 [49]	1	1	0	1	1	1	1	6
Parkar, 2016 [50]	1	1	0	1	1	1	1	6
Plummer, 2011 [51]	1	0	0	1	1	1	1	5
Sangrasi, 2014 [52]	1	0	0	1	0	1	1	4
Shehata, 2012 [53]	1	0	0	1	1	1	1	5
Tayeb, 2008 [54]	1	0	0	1	0	1	1	4
Wani, 2018 [55]	1	0	0	1	1	1	1	5

References

1. Abro AH, Pathan AH, Dal NA, Laghari AA, Abbasi KH, Qasim S, Durrani J. Demo- graphical Evaluation of Laparoscopic Versus Open Appendectomy at Tertiary Care Teaching Hospital. J Liaquat Uni Med Health Sci. 2017;16(01):24-8.
2. Adisa AO, Alatise OI, Arowolo OA, Lawal OO. Laparoscopic appendectomy in a Nigerian teaching hospital. JSLS. 2012 Oct-Dec;16(4):576-80.
3. Akkary R, Zeidan S, Matta R, Lakis C, Diab N. Pediatric appendectomy in developing countries: How does it differ from international experience? Int J Pediatr Adolesc Med. 2020 Jun;7(2):70-73.
4. Ali R, Khan MR, Pishori T, Tayeb M. Laparoscopic appendectomy for acute appendicitis: Is this a feasible option for developing countries? Saudi J Gastroenterol. 2010 Jan-Mar;16(1):25-9.
5. Ali R, Anwar M, Akhtar J. Laparoscopic versus open appendectomy in children: a randomized controlled trial from a developing country. J Pediatr Surg. 2018 Feb;53(2):247-249.
6. Bedada AG, Hsiao M, Bakanisi B, Motsumi M, Azzie G. Establishing a contextually appropriate laparoscopic program in resource-restricted environments: experience in Botswana. Ann Surg. 2015 Apr;261(4):807-11.
7. Cakmak Y, Comert DK, Sozen I, Oge T. Comparison of Laparoscopy and Laparotomy in Early-Stage Endometrial Cancer: Early Experiences from a Developing Country. J Oncol. 2020 Apr 30;2020:2157520.
8. Cao JG, Tao F, Zhou XJ et al. Trends and outcomes of laparoscopic appendectomy in China: a multicenter, retrospective cohort study. Surg. Pract. 2015; 19: 166–72.
9. Chen B, Hu S, Wang L, Wang K, Zhang G, Zhang H et al. Economical Strategies of Laparoscopic Splenectomy: A Chinese Single-Center Experience. Chir Gastroenterol 2007;23:387–391
10. Darwish AM, Amin AF, AbdelAleem MA, Youssef MA. Laparoscopic management of benign adnexal masses: experience in a developing country. Gynaecological Endoscopy 2001 10, 159±165.
11. Hamamci EO, Besim H, Bostanoglu S, Sonişik M, Korkmaz A. Use of laparoscopic splenectomy in developing countries: analysis of cost and strategies for reducing cost. J Laparoendosc Adv Surg Tech A. 2002 Aug;12(4):253-8.
12. Khalil J, Muqim R, Rafique M, Khan M. Laparoscopic versus open appendectomy: a comparison of primary outcome measures. Saudi J Gastroenterol. 2011 Jul-Aug;17(4):236-40.
13. Kumar A, Dubey D, Gogoi S, Arvind NK. Laparoscopy-assisted live donor nephrectomy: a modified cost-effective approach for developing countries. J Endourol. 2002 Apr;16(3):155-9.
14. Kumar A, Agrahari A, Pahwa HS, Anand A, Singh S, Kushwaha JK, Sonkar AA. A Prospective Nonrandomized Study of Comparison of Perioperative and Quality of Life Outcomes of Endoscopic Versus Open Inguinal Hernia Repair: Data from a Developing Country. J Laparoendosc Adv Surg Tech A. 2017 Mar;27(3):264-267.
15. Lu Z, Yi X, Feng W, Ding J, Xu H, Zhou X, Hua K. Cost-benefit analysis of laparoscopic surgery versus laparotomy for patients with endometrioid endometrial cancer: experience from an institute in China. J Obstet Gynaecol Res. 2012 Jul;38(7):1011-7.
16. Mahadevappa K, Prasanna N, Channabasappa RA. Trends of Various Techniques of Tubectomy: A Five Year Study in a Tertiary Institute. J Clin Diagn Res. 2016 Jan;10(1):QC04-7.
17. Mahmoud AMA, Moneer MM. Toward standardization of laparoscopic resection for colorectal cancer in developing countries: A step by step module. J Egypt Natl Canc Inst. 2017 Sep;29(3):135-140.
18. Manning RG, Aziz AQ. Should laparoscopic cholecystectomy be practiced in the developing world?: the experience of the first training program in Afghanistan. Ann Surg. 2009 May;249(5):794-8.
19. Ruiz-Patiño A, Rey S, Molina G, Dominguez LC, Rugeles S. Cost-effectiveness of laparoscopic versus open appendectomy in developing nations: a Colombian analysis. J Surg Res. 2018 Apr;224:33-37.
20. Shaikh AR, Sangrasi AK, Shaikh GA. Clinical outcomes of laparoscopic versus open appendectomy. JSLS. 2009 Oct-Dec;13(4):574-80.
21. Tunruttanakul S, Chareonsil B, Charernsuk M. Operative outcome of laparoscopic colorectal cancer surgery in a regional hospital in a developing country: A propensity score-matched comparative analysis. Asian J Surg. 2021 Jan;44(1):329-333.
22. Amin AT, Ahmed BM, Khallaf SM. Safety and feasibility of laparoscopic colo-rectal surgery for cancer at a tertiary center in a developing country: Egypt as an example. J Egypt Natl Canc Inst. 2015 Jun;27(2):91-5.

23. Aslam MN, Butt F, Ashraf S. An experience of Laparoscopic Cholecystectomy from Secondary Care Center of Pakistan.P J M H S Vol. 11, NO. 1, 2017 105-108.
24. Ayandipo O, Afuwape O, Olonisakin R. Laparoscopic cholecystectomy in ibadan, southwest Nigeria. J West Afr Coll Surg. 2013 Apr;3(2):15-26.
25. Bal S, Reddy LG, Parshad R, Guleria R, Kashyap L. Feasibility and safety of day care laparoscopic cholecystectomy in a developing country. Postgrad Med J. 2003 May;79(931):284-8.
26. Balogun OS, Osinowo AO, Olajide TO, Lawal AO, Adesanya AA, Atoyebi OA, et al. Development and practice of laparoscopic surgery in a Nigerian tertiary hospital. Niger J Clin Pract 2020;23:1368-74.
27. Benkabbou A, Souadka A, Serji B, Hachim H, El Malki HO, Mohsine R, Ifrine L, Belkouchi A. Laparoscopic liver resection: initial experience in a North-African single center. Tunis Med. 2015 Aug-Sep;93(8-9):523-6.
28. Cawich SO, Mohanty SK, Simpson LK, Bonadie KO. Is emergent laparoscopic cholecystectomy for acute cholecystitis safe in a low volume resource poor setting? Int J Surg. 2014;12(8):798-802.
29. Chauhan A, Mehrotra M, Bhatia PK, Baj B, Gupta AK. Day care laparoscopic cholecystectomy: a feasibility study in a public health service hospital in a developing country. World J Surg. 2006 Sep;30(9):1690-5; discussion 1696-7.
30. Dalvi AN, Thapar PM, Deshpande AA, Rege SA, Prabhu RY, Supe AN, Kamble RS. Laparoscopic splenectomy using conventional instruments. J Minim Access Surg. 2005 Jun;1(2):63-9.
31. Dan D, Harnanan D, Gooden K, Seetahal S, Hariharan S, Naraynsingh V. Laparoscopic splenectomy in a minimal resource setting: a case series from the Caribbean. Surg Laparosc Endosc Percutan Tech. 2010 Jun;20(3):e125-8.
32. Farooq U, Rashid T, Naheed A, Barkat N, Iqbal M, Sultana Q. COMPLICATIONS OF LAPAROSCOPIC CHOLECYSTECTOMY: AN EXPERIENCE OF 247 CASES. J Ayub Med Coll Abbottabad. 2015 Apr-Jun;27(2):407-10.
33. Gupta V, Hatimi H, Kumar S, Chandra A. Laparoscopic Heller's Myotomy for Achalasia Cardia: One-Time Treatment in Developing Countries? Indian J Surg. 2017 Oct;79(5):401-405. doi: 10.1007/s12262-016-1496-8.
34. Hannan MJ, Hoque MM, Begum LN. Laparoscopic appendectomy in pregnant women: experience in Chittagong, Bangladesh. World J Surg. 2012 Apr;36(4):767-70.
35. Ismail S, Ahmed A, Hoda MQ, Sohaib M, Zia-Ur-Rehman. Prospective survey to study factors which could influence same-day discharge after elective laparoscopic cholecystectomy in a tertiary care hospital of a developing country. Updates Surg. 2016 Dec;68(4):387-393.
36. Jategaonkar PA, Yadav SP. Trans-umbilical Laparoscopic Appendectomy for Acute Appendicitis without Raising Skin-flaps: An Easy-to-use Modification Applied to the Series of 164 Patients from a Rural Institute of Central India. J Surg Tech Case Rep. 2013 Jan;5(1):8-12.
37. Karim T, Katiyar VK, Singh R, Dey S. Short-term Results of Laparoscopic Transabdominal Preperitoneal Inguinal Hernioplasty in a Developing Country. World J Lap Surg 2017; 10 (3):83-86.
38. Kumar A, Chaudhary H, Srivastava A, Raghavendran M. Laparoscopic live-donor nephrectomy: modifications for developing nations. BJU Int. 2004 Jun;93(9):1291-5.
39. Kumar A, Gupta NP, Hemal AK. A single institution experience of 141 cases of laparoscopic radical nephrectomy with cost-reductive measures. J Endourol. 2009 Mar;23(3):445-9.
40. Mahdi BD, Rahma C, Mohamed J, Riadh M. Single-port laparoscopic surgery in children: A new alternative in developing countries. Afr J Paediatr Surg. 2015 Apr-Jun;12(2):122-5.
41. Mboudou E, Morfaw FL, Foumane P, Sama JD, Mbatsogo BA, Minkande JZ. Gynaecological laparoscopic surgery: eight years experience in the Yaoundé Gynaeco-Obstetric and Paediatric Hospital, Cameroon. Trop Doct. 2014 Apr;44(2):71-6.
42. Mir IS, Mohsin M, Kirmani O, Majid T, Wani K, Hassan MU, Naqshbandi J, Maqbool M. Is intra-operative cholangiography necessary during laparoscopic cholecystectomy? A multicentre rural experience from a developing world country. World J Gastroenterol. 2007 Sep 7;13(33):4493-7.
43. Mir IS, Mohsin M, Kirmani O, Cheachek BA, Alam I, Wani M. Is laparoscospic orchidectomy the treatment of choice in adults with impalpable testis in rural hospitals in the developing world? Trop Doct. 2009 Jan;39(1):12-5.
44. Misauno M A, Ojo E O, Uba A F. Laparoscopic paediatric surgery: A potential for paradigm shift in developing countries.Afr J Paediatr Surg 2012;9:140-142
45. Mishra A, Bains L, Jesudin G, Aruparayil N, Singh R, Shashi. Evaluation of Gasless Laparoscopy as a Tool for Minimal Access Surgery in Low-to Middle-Income Countries: A Phase II Noninferiority Randomized Controlled Study. J Am Coll Surg. 2020 Nov;231(5):511-519.
46. Mucio M, Felemovicius J, De la Concha F, Cabello R, Zamora A. The Mexican experience with laparoscopic cholecystectomy and common bile duct exploration. A multicentric trial. Surg Endosc. 1994 Apr;8(4):306-9.
47. Nande AG, Shrikhande SV, Rathod V, Adyanthaya K, Shrikhande VN. Modified technique of gasless laparoscopic cholecystectomy in a developing country: a 5-year experience. Dig Surg. 2002;19(5):366-71.
48. O'Connor Z, Faniriko M, Thelander K, O'Connor J, Thompson D, Park A. Laparoscopy Using Room Air Insufflation in a Rural African Jungle Hospital: The Bongolo Hospital Experience, January 2006 to December 2013. Surg Innov. 2017 Jun;24(3):264-267.
49. Pareja R, Nick AM, Schmeler KM, Frumovitz M, Soliman PT, Buitrago CA, Borrero M, Angel G, Reis RD, Ramirez PT. Quality of laparoscopic radical hysterectomy in developing countries: a comparison of surgical and oncologic outcomes between a comprehensive cancer center in the United States and a cancer center in Colombia. Gynecol Oncol. 2012 May;125(2):326-9.
50. Parkar RB, Pinder LF, Wanyoike J, Patel Y, Otieno D, Palkhi Y, Baraza R, Rogo K. Laparoscopic Surgery in Low-income and Limited-resource Settings: Does It safely add Value? A Review of 2,901 Laparoscopic Gynecologic Procedures. World J Lap Surg 2016; 9 (2):82-85.

51. Plummer JM, Mitchell DI, Arthurs M, Leake PA, Deans-Minott J, Cawich SO, Martin A. Laparoscopic colectomy for colonic neoplasms in a developing country. Int J Surg. 2011;9(5):382-5.
52. Sangrasi AK, Syed B, Memon AI, Laghari AA, Talpur KA, Qureshi JN. Laparoscopic cholecystectomy in acute gallstone pancreatitis in index hospital admission: feasibility and safety. Pak J Med Sci. 2014 May;30(3):601-5.
53. Shehata SM, El Attar AA, Attia MA, Hassan AM. Laparoscopic herniotomy in children: prospective assessment of tertiary center experience in a developing country. Hernia. 2013 Apr;17(2):229-34.
54. Tayeb M, Khan MR, Riaz N. Laparoscopic cholecystectomy in cirrhotic patients: feasibility in a developing country. Saudi J Gastroenterol. 2008 Apr;14(2):66-9.
55. Wani MM, Durrani AM. Laparoscopic ureterolithotomy: Experience of 60 cases from a developing world hospital. J Minim Access Surg. 2018 May 4;15(2):103–8.
